# A Scorpion Defensin BmKDfsin4 Inhibits Hepatitis B Virus Replication *in Vitro*

**DOI:** 10.3390/toxins8050124

**Published:** 2016-04-27

**Authors:** Zhengyang Zeng, Qian Zhang, Wei Hong, Yingqiu Xie, Yun Liu, Wenxin Li, Yingliang Wu, Zhijian Cao

**Affiliations:** 1 State Key Laboratory of Virology, College of Life Sciences, Wuhan University, Wuhan 430072, China; zzybio@126.com (Z.Z.); white2459533@163.com (Q.Z.); whong1@partners.org (W.H.); 2013301580085@whu.edu.cn (Y.L.); liwxlab@whu.edu.cn (W.L.); ylwu@whu.edu.cn (Y.W.); 2 Department of Biology, Nazarbayev University School of Science and Technology, Astana 010000, Republic of Kazakhstan; yingqiu.xie@nu.edu.kz

**Keywords:** HBV, scorpion defensin, antiviral peptide, multifunction

## Abstract

Hepatitis B virus (HBV) infection is a major worldwide health problem which can cause acute and chronic hepatitis and can significantly increase the risk of liver cirrhosis and primary hepatocellular carcinoma (HCC). Nowadays, clinical therapies of HBV infection still mainly rely on nucleotide analogs and interferons, the usage of which is limited by drug-resistant mutation or side effects. Defensins had been reported to effectively inhibit the proliferation of bacteria, fungi, parasites and viruses. Here, we screened the anti-HBV activity of 25 scorpion-derived peptides most recently characterized by our group. Through evaluating anti-HBV activity and cytotoxicity, we found that BmKDfsin4, a scorpion defensin with antibacterial and Kv1.3-blocking activities, has a comparable high inhibitory rate of both HBeAg and HBsAg in HepG2.2.15 culture medium and low cytotoxicity to HepG2.2.15. Then, our experimental results further showed that BmKDfsin4 can dose-dependently decrease the production of HBV DNA and HBV viral proteins in both culture medium and cell lysate. Interestingly, BmKDfsin4 exerted high serum stability. Together, this study indicates that the scorpion defensin BmKDfsin4 also has inhibitory activity against HBV replication along with its antibacterial and potassium ion channel Kv1.3-blocking activities, which shows that BmKDfsin4 is a uniquely multifunctional defensin molecule. Our work also provides a good molecule material which will be used to investigate the link or relationship of its antiviral, antibacterial and ion channel–modulating activities in the future.

## 1. Introduction

Hepatitis B virus (HBV) is the prototypic member of hepadnaviridae and the pathogenic cause of acute and chronic type B hepatitis in humans. Chronic HBV infection, which now affects health of more than 400 million people around the world, has been demonstrated to result in a 100-fold elevation in the risk of developing hepatocellular carcinoma (HCC) [1]. Although effective vaccines have been extensively applied for years, the increase in the amount of infected individuals and the difficulties of treatment after infection are still perplexing to physicians and researchers. Interferon-α and nucleotide analogues are available for alleviating a patient’s condition, but their therapeutic application is seriously limited by side effects and resistant mutated virus strains. Furthermore, combination therapies based on these drugs exerted unsatisfactory response efficiency [2,3]. Therefore, studies aimed at developing new, efficient anti-HBV agents are very urgently desired.

Scorpions evolved particular venom systems during their hundreds of millions of years of evolution. For the sake of predation and defending predators, multiple types of neurotoxins have been generated, which can block or regulate the activities of a variety of ion channels [4,5,6,7]. In addition, anti-microbial peptides (AMPs) are also major components of scorpion venom peptides. These small peptides have exerted functional diversity, such as modulating innate immunity, regulating oncogenesis and directly inhibiting various bacteria, viruses, fungi and parasites [8,9,10]. In recent years, several studies about scorpion-derived antiviral peptides have been reported. For example, a scorpion venom–derived synthetic peptide, Mucroporin-M1, was reported to immediately interfere the virion of measles, SARS-CoV and influenza H5N1 viruses [11], and inhibit HBV replication *in vitro* and *in vivo* by activating the mitogen-activated protein kinase (MAPK) pathway and down-regulating HNF4α [10]. Mutations of another scorpion-derived peptide, Ctry2459, showed an extremely enhanced bioavailability and anti-HCV activity compared with the wild-type peptide [12]. In addition, another two scorpion venom peptides were also reported due to their inhibitory activity against herpes simplex virus type I [13]. Thus, scorpion venom is a resource of novel antiviral peptides.

In this study, we present a scorpion defensin BmKDfsin4, derived from the scorpion *Mesobuthus martensii* Karsch, which effectively inhibited HBV replication *in vitro*. BmKDfsin4 stood out from 25 scorpion peptides because of its high inhibition rate of HBeAg and HBsAg in the supernatant of HepG2.2.15 cells and relatively low cytotoxicity. Then, a further investigation of cytotoxicity and anti-HBV activity of BmKDfsin4 had been conducted which concluded that the inhibitory activity of BmKDfsin4 against HBV replication is concentration-dependent and its selective index (ratio of CC_50_ to IC_50_) is relatively high compared with its cytotoxicity. Furthermore, BmKDfsin4 also exerted considerable serum stability. BmKDfsin4 is the first reported scorpion defensin, which is a novel anti-HBV agent with potential for future modification and application.

## 2. Results

### 2.1. Screening of Anti-HBV Agents from Scorpion-Derived Peptides

**Figure 1:**
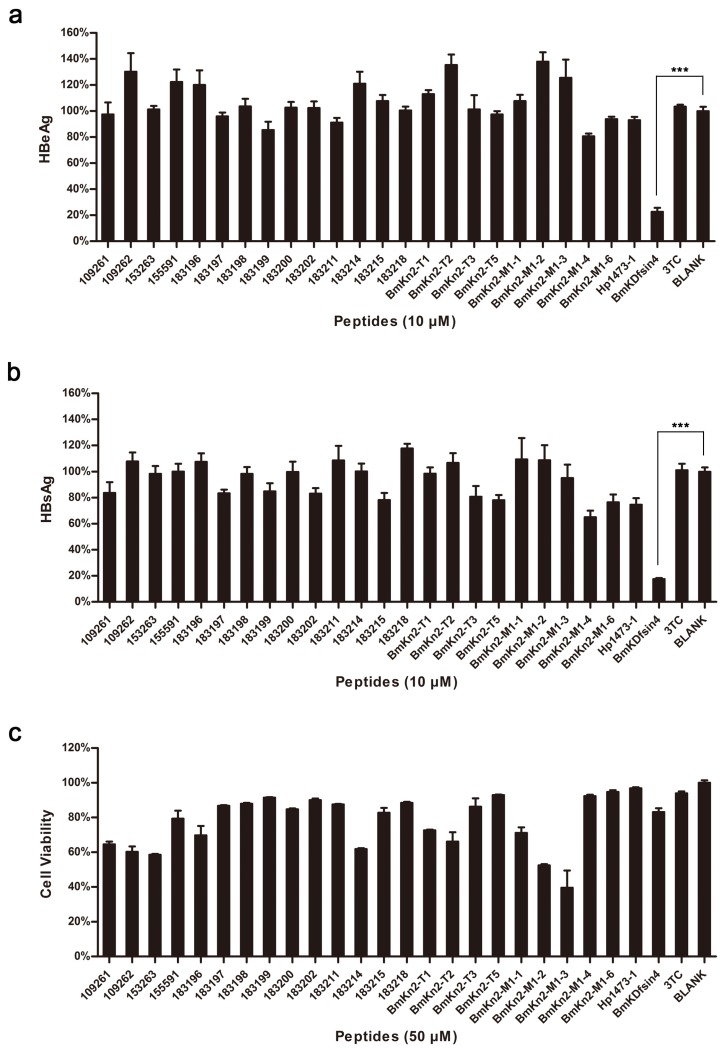
Screening of anti-HBV peptides from the scorpion venom peptide libraries. Fifteen scorpion peptides from the venomous cDNA libraries of four scorpion species (*Mesobuthus martensii*, *Heterometrus petersii*, *Chaerilus tricostatus* and *Chaerilus tryznai*) and 10 derivative peptides were characterized as candidates of antimicrobial agents. They were prepared by prokaryotic expression system or chemical synthesis. (**a**,**b**) Twenty-five peptides (10 μM) were used to screen the inhibitory ability against HBV by ELISA assays of HBeAg and HBsAg in the culture medium of HepG2.2.15 cells. The average inhibitory rate of BmKDfsin4 against HBeAg and HBsAg at 10 μM was 77.46% and 82.46%, respectively; (**c**) Cytotoxicity of these 25 peptides at 50 μM was measured by an MTT assay. The cell viability of HepG2.2.15 cells after being treated with 50 μM BmKDfsin4 for 48 h was 83.18%.*** denotes *p* < 0.001. Values represent the mean ± SEM of five independent samples.

Fifteen putative peptides from the venomous cDNA libraries of four scorpion species (*Mesobuthus martensii*, *Heterometrus petersii, Chaerilus tricostatus* and *Chaerilus tryznai*) and 10 derivative peptides were identified as candidate antimicrobial agents and were prepared by the prokaryotic expression technique [14,15] or chemical synthesis [10,12,13] (Table S1). The anti-HBV activity of these peptides at 10 μM was then determined by measuring the concentrations of HBeAg (Figure 1a) and HBsAg (Figure 1b) in cell culture medium of HepG2.2.15 cells by ELISA assays. BmKDfsin4 exerted a higher inhibitory activity against both HBeAg and HBsAg compared with any other scorpion-derived peptide. The inhibitory effect was 77.46% and 82.46%, respectively. The cytotoxicity of these peptides at 50 μM on HepG2.2.15 cells was measured by an MTT assay (Figure 1c). After being treated with BmKDfsin4 at 50 μM for 48 h, the cell viability of HepG2.2.15 cells was 83.18%, which indicates a relatively low cytotoxicity.

### 2.2. Cytotoxicity and Hemolysis of BmKDfsin4

**Figure 2:**
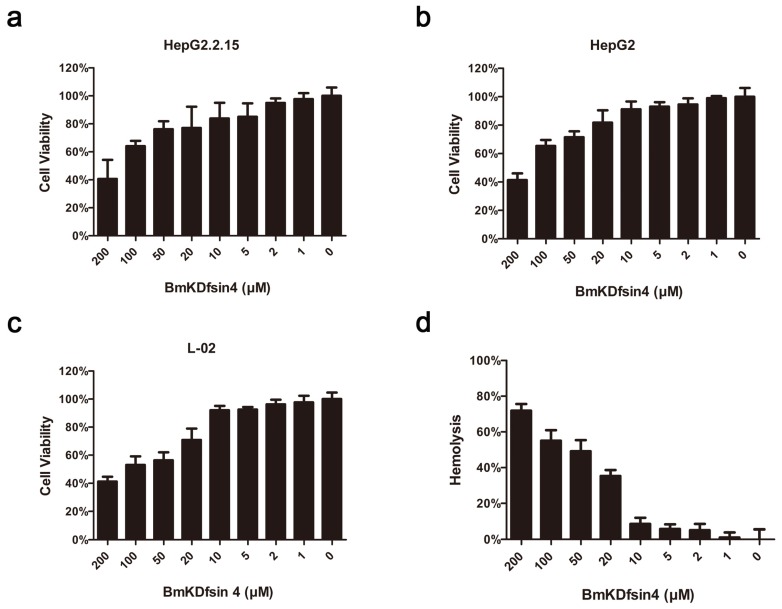
Cytotoxicity of BmKDfsin4 on hepatic cells and its hemolysis activity*.* The cytotoxicity of BmKDfsin4 was measured on HepG2.2.15 (**a**); HepG2 (**b**) and L-02 (**c**) cells using MTT assays. The 50% cytotoxicity concentrations (CC_50_) of BmKDfsin4 on HepG2.2.15, HepG2 and L-02 were 167.82, 154.24 and 103.77 μM, respectively; (**d**) The hemolytic activity of BmKDfsin4 was evaluated in human erythrocytes using hemolytic assay. The 50% hemolysis concentration was 66.85 μM. The concentrations ranged from 0 to 200 μM. Values represent the mean ± SEM of five independent samples.

The feasibility of the further development of BmKDfsin4 as a candidate anti-HBV agent was determined by measuring its cytotoxicity. After being incubated with a serial dilution of BmKDfsin4 for 48 h, the cell viability of HepG2.2.15 (Figure 2a), HepG2 (Figure 2b) and L-02 (Figure 2c) cells was measured using MTT assays. The 50% cytotoxicity concentrations (CC_50_) of BmKDfsin4 to HepG2.2.15, HepG2 and L-02 were 167.82, 154.24 and 103.77 μM, respectively. At the concentration of 10 μM, the viability of the BmKDfsin4-treated cells was greater than 90% in all three kinds of cell lines. The hemolysis assay also showed that the viability of erythrocytes was more than 90% when the concentration of BmKDfsin4 was lower than 10 μM (Figure 2d). These data indicated that 10 μM or less BmKDfsin4 was minimally cytotoxic and suitable for further anti-HBV studies.

### 2.3. Extracellular Anti-HBV Activity of BmKDfsin4 in Vitro

**Figure 3:**
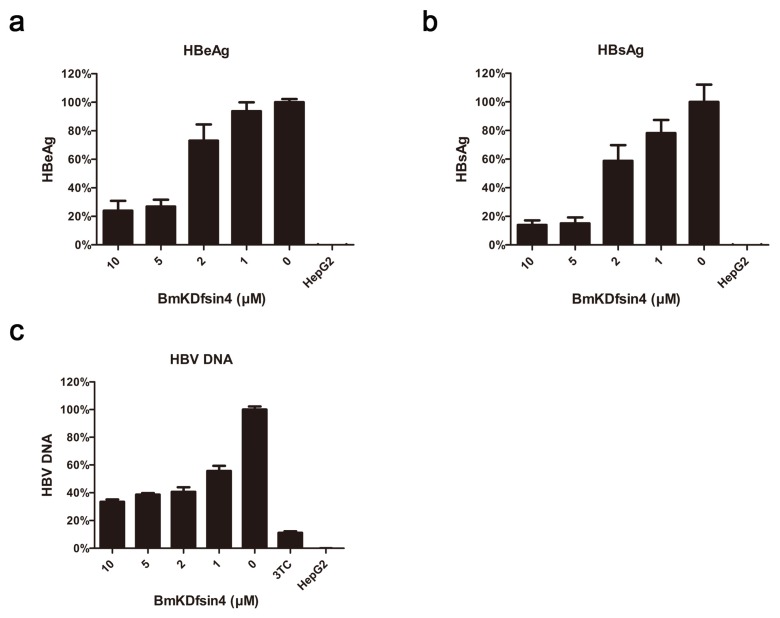
Extracellular anti-HBV activity of BmKDfsin4 in HepG2.2.15 cells. (**a**,**b**) The inhibitory effect of BmKDfsin4 against HBeAg and HBsAg in HepG2.2.15 culture medium was measured using ELISA. The 50% inhibitory concentrations (IC_50_) were 3.95 and 2.28 μM, respectively; (**c**) The inhibitory effect of BmKDfsin4 against HBV DNA in HepG2.2.15 culture medium was measured using real-time PCR. The 50% inhibitory concentration (IC_50_) of BmKDfsin4 against HBV DNA was 1.26 μM. The drug 3TC was used as a control. Values represent the mean ± SEM of five independent samples.

To further investigate the effect of BmKDfsin4 on HBV replication *in vitro*, HepG2.2.15 cells were treated with a serial dilution of BmKDfsin4 for 48 h. HBeAg and HBsAg levels in HepG2.2.15 cell supernatant after being treated with BmKDfsin4 were measured by ELISA assays. As shown in Figure 3a,b, the cellular supernatant HBeAg and HBsAg levels were significantly reduced by BmKDfsin4 in a dose-dependent manner. The 50% inhibitory concentrations (IC_50_) were 3.95 and 2.28 μM, yielding selective indexes (ratio of CC_50_ to IC_50_) as 42.78 and 74.11, respectively (Table 1). HBV progeny DNA in the supernatant of HepG2.2.15 cells was quantitated using real-time PCR. Real-time PCR data indicated that HBV progeny DNA in the culture media was also inhibited by BmKDfsin4 in a concentration-dependent manner (IC_50_ = 1.26 μM) (Figure 3c) and the selective index was 133.20 (Table 1). Thus, BmKDfsin4 can reduce levels of both HBV antigens and HBV progeny DNA in HepG2.2.15 cell culture supernatant in a concentration-dependent manner.

### 2.4. Intracellular Anti-HBV Activity of BmKDfsin4 in Vitro

**Figure 4:**
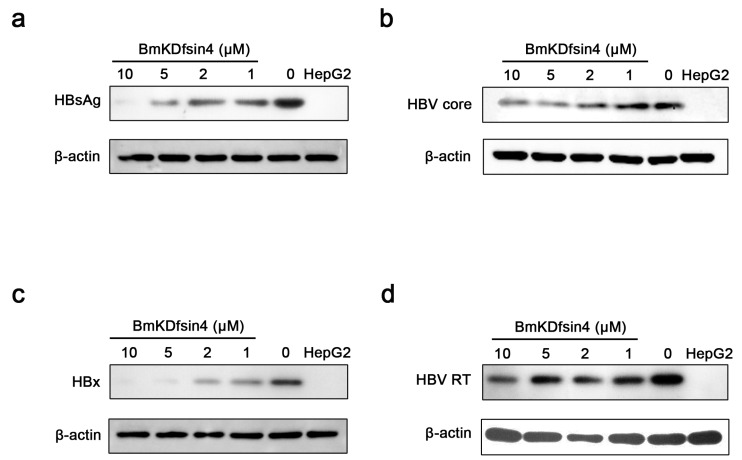
Intracellular anti-HBV activity of BmKDfsin4 in HepG2.2.15 cells. (**a**–**d**) The inhibitory effects of BmKDfsin4 against HBsAg, HBV core, HBx, and HBV RT proteins in HepG2.2.15 cell lysates were determined using Western blot analysis. HepG2 cells were used as negative control.

The levels of intracellular HBV viral proteins were measured by Western blot analysis. Similar to the effects in HepG2.2.15 cell culture supernatant, HepG2.2.15 intracellular HBsAg (Figure 4a) was also inhibited by BmKDfsin4 in a dose-dependent manner. When treated by BmKDfsin4 at 10 μM, HBsAg was almost undetectable. BmKDfsin4 seems to have extremely strong inhibitory activity against HBx protein (Figure 4c). HepG2.2.15 cells treated by BmKDfsin4 at 5 μM had only a slight band of HBx protein, while the HBx protein of HepG2.2.15 cells was totally undetectable after the treatment of 10 μM BmKDfsin4. The inhibitory activity of BmKDfsin4 against HBV core (Figure 4b) and HBV RT (Figure 4d) seems lower compared with HBsAg and HBx protein, but it still exerted a significantly concentration-dependent inhibition. These results suggest that BmKDfsin4 has an inhibitory activity against intracellular HBV viral proteins at minimally cytotoxic concentrations and these inhibitory effects were dose-dependent.

### 2.5. Serum Stability of BmKDfsin4

**Figure 5:**
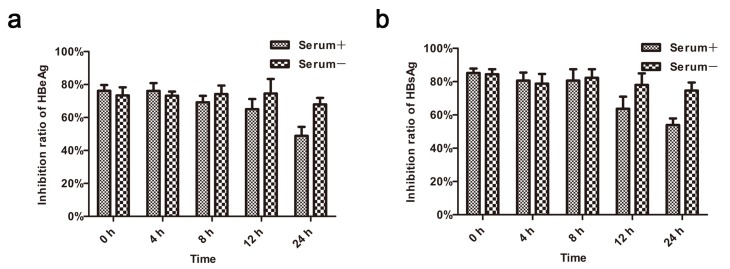
Serum stability of BmKDfsin4. (**a**,**b**) BmKDfsin4 was incubated in culture medium containing 10% FBS for 0, 4, 8, 12 and 24 h at 37 °C. At the indicated time points, pre-incubated BmKDfsin4 aliquots were used for antiviral assays. The levels of HBeAg and HBsAg in HepG2.2.15 culture medium were measured using ELISA. Values represent the mean ± SEM of five independent samples.

The serum stability of BmKDfsin4 was tested by inhibitory activity against HBeAg and HBsAg in the supernatant of HepG2.2.15 at 10 μM after incubation in 10% FBS for less than 24 h. After being incubated with 10% serum for 8 h or less, the inhibitory rate of BmKDfsin4 against HBeAg and HBsAg was similar to the rate of the serum-untreated BmKDfsin4. There was a slight decrease of inhibitory ratio after being treated with 10% serum for 12 and 24 h, but the inhibitory ratio of BmKDfsin4 against HBeAg was still 65.01% and 48.85%, respectively (Figure 5a). Meanwhile, the inhibitory ratio of BmKDfsin4 against HBsAg was 63.69% and 54.00%, respectively (Figure 5b). These results suggest that BmKDfsin4 has a high serum stability.

## 3. Discussion

The application of interferons is limited by their side effects, and nucleotide analog treatments such as lamivudine (3TC) have the potential of inducing mutations in viral polymerase, which can prompt drug-resistant virus strains. Thus, the demand for the development of new potential anti-HBV drugs is still urgent. In recent years, many newly discovered or designed anti-HBV compounds or organic molecules have been reported. Cyclosporin A was reported to inhibit HBV and HDV at the entry stage by interfering with NTCP (sodium taurocholate co-transporting polypeptide) [16]. Acylated peptides derived from the large viral surface protein inhibited HBV infection with an extremely low IC_50_ [17]. 

Insect defensins are small cationic peptides composed by 34–51 amino acid residues, which commonly contain six cysteines. The broad spectrum antimicrobial activity of insect defensins involves bacteria [14,18,19,20,21,22,23], some fungi [20,21,22,24,25,26] and even parasites [27,28]. Several scorpion defensins or defensin-like antimicrobial peptides have been discovered. A 4 kDa scorpion defensin was purified in 1993, which presents structural similarity with insect defensins and scorpion toxins [29]. Another 4 kDa scorpion defensin (SD) was expressed in yeast and its *in vitro* synergistic activity with conventional antibiotics was identified [30].

Our experimental results showed that BmKDfsin4 exerted considerable inhibitory activity on HBV replication by decreasing the production of HBeAg (IC_50_ = 3.95 μM), HBsAg (IC_50_ = 2.28 μM) and HBV DNA (IC_50_ = 1.26 μM) in cell culture medium, and the production of intracellular HBsAg, HBV core protein, HBx protein and HBV RT and the inhibition were concentration-dependent. Compared with its high anti-HBV activity, the MTT assay indicated that BmKDfsin4 has a low cytotoxicity in HepG2.2.15 cells (CC_50_ = 167.82 μM), HepG2 cells (CC_50_ = 154.24 μM) and L-02 cells (CC_50_ = 103.77 μM). As for HepG2.2.15 cells, which is a stable transferred cell line of 1.3-fold HBV genome, the selective index of BmKDfsin4 was valued as high as 133.20. In addition, BmKDfsin4 also exerted extremely low hemolytic activity on human erythrocytes. The serum stability of BmKDfsin4 was measured by the inhibitory rate of HBeAg and HBsAg, after being incubated with 10% serum for a serial time points. As there was only a slight decrease in inhibitory activity against both HBeAg and HBsAg after a 24 h incubation, BmKDfsin4 was thought to have a strong serum stability.

To the best of our knowledge, all reported antiviral peptides derived from scorpion venom were short-chain polypeptides with 13–20 amino acid residues and without any disulfide bridge [10,11,12,13,31]. Having the typical sequence and predicted structure characteristics of defensin, BmKDfsin4 is the first scorpion defensin with experimental evidence of antiviral activity. It is worth mentioning that only the scorpion defensin BmKDfsin4 can inhibit HBV replication in the cultured HepG2.2.15 system among the tested 25 peptides from scorpion venoms. Different from BmKDfsin4, all the other 24 non-active peptides are short linear peptides which are not cross-linked by disulfide bridges. The scorpion defensin BmKDfsin4 should be deduced to have more strong serum stability than the other 24 short linear peptides, which was partially confirmed by the result that BmKDfsin4 has a strong serum stability in the above anti-HBV tests. Meanwhile, the phenomenon was possibly related to antiviral mechanisms. Although the antiviral mechanisms of linear peptides are various, most short linear peptides may inhibit virus infection and replication by interacting with viral envelopes or surfaces of capsids and then destroying the structure of virions. However, HepG2.2.15, the cell line used for the antiviral experiments in our study, was derived from a human hepatoblastoma (HepG2). In HepG2.2.15, HBV genome was stably incorporated into the host genome and HBV was replicated. Therefore, HepG2.2.15 could continuously produce infectious HBV virions, but it was not successfully infected by HBV particles.

In addition, most recently, our group had also reported that BmKDfsin4 can effectively and selectively inhibit the growth of Gram-positive bacteria, including some standard and antibiotic-resistant strains, and block the potassium channel with a neurotoxin-like pore-blocking mode. [14] Thus, BmKDfsin4 is the first reported multi-functional defensin with three different aspects of bio-activities. Whether there is an internal relation between its three biological functions deserves further research and exploration in the future.

## 4. Materials and Methods

### 4.1. Reagents

BmKDfsin4 was expressed using the prokaryotic expressing system of our laboratory. Other peptides were synthesized using the solid-phase synthesis method and amidated at the *C*-terminus with a purity of >95% (ChinaPeptides Co., Ltd., Shanghai, China). All peptides were assessed by HPLC (Elite-HPLC, Los Angeles, CA, USA) and mass spectrometry (Voyager-DESTR; Applied Biosystems, Foster, CA, USA). The positive drug control lamivudine (3TC), the penicillin and streptomycin used in cell cultures and the dimethylsulfoxide (DMSO) used for MTT assay were all purchased from Sigma (Sigma, St. Louis, MO, USA). The dose of 3TC used for the anti-HBV activity experiments was 20 μM. Real-time PCR primers were synthesized by Sangon Biological Engineering Technology Company (Shanghai, China).

### 4.2. Cell Culture

Dulbecco’s modified Eagle’s medium (DMEM) (Invitrogen, Foster, CA, USA) supplemented with 10% (*vol*/*vol*) fetal bovine serum (FBS) (Gibco, Foster, CA, USA), 50 units/mL penicillin and 50 μg/mL streptomycin was used to culture all three cell lines. Cells were cultured at 37 °C in a 5% carbon dioxide incubator.

### 4.3. MTT Assay

Cells were seeded in 96-well plates (10^4^ cells per well) and cultured in DMEM containing 10% FBS in 5% CO_2_ at 37 °C for 12 h. Then, peptides were added to the medium in a serial of concentrations. The concentrations ranged from 0 to 200 μM. Five repetitions were set for each concentration. After 48 h of incubation, 20 μL of MTT solution (5 mg/mL in PBS buffer; Invitrogen, Foster, CA, USA) was added to the medium in each well, and the plate was incubated in 5% CO_2_ at 37 °C for 4 h. Then, the medium was replaced by 100 μL DMSO. To completely dissolve the precipitated crystal purple formazan, the plate was then gently shaken for 10 min at room temperature. The absorbance at a wavelength of 570 nm was measured using a microplate reader (BioTek, Winooski, VT, USA).

### 4.4. Hemolysis Assay

Erythrocytes were obtained from fresh human blood by centrifugation for 10 min at 1000× *g*. After washed by HEPES buffer for three times, erythrocytes were resuspended in normal saline and seeded in 96-well plates (10^7^ cells per well). BmKDfsin4 was added in a serial of concentrations, 0.1% triton-X100 was used as positive control and normal saline was used as negative control. After incubated at 37 °C for 1 h, the supernatants were collected by centrifugation for 10 min at 1000× *g*. The absorbance of hemoglobin was measured at a wavelength of 570 nm.

### 4.5. Quantification of HBeAg, HBsAg and HBV DNA in the Culture Medium

HepG2.2.15 cells were seeded in 24-well plates (5 × 10^4^ cells per well) and incubated at 37 °C for 12 h. Then, the cells were treated with various concentrations of BmKDfsin4 and cultured for another 48 h. HBeAg and HBsAg levels were quantitated using an enzyme linked immunoassay (ELISA) kit. HBV DNA in the culture medium was extracted and measured using real-time PCR (QIAGEN, Valencia, CA, USA).

### 4.6. Western Blot Analysis

Cells were resuspended with radioimmunoprecipitation (RIPA) assay buffer (50 mM Tris-HCl (pH 7.5), 150 mM sodium chloride, 1% Nonidet P40, and 0.5% sodium deoxycholate) with phenylmethylsulfonyl fluoride (PMSF) and incubated on ice for 30 min for fully lysis. Cell lysates were electrophoresed in sodium dodecyl sulfate-polyacrylamide (SDS) gels and transferred to nitrocellulose membranes (Millipore, Darmstadt, Germany). The antibodies used for probing were as follows: mouse monoclonal anti-HBsAg (1:200 dilution, Abcam, Cambridge, UK), rabbit polyclonal anti-HBV core (1:500 dilution, Dako-Cytomation, Carpinteria, CA, USA), mouse monoclonal anti-Hep B xAg (1:200 dilution, Santa Cruz Biotechnology, Dallas, TX, USA), mouse monoclonal anti-Hep B Pol (1:200 dilution, Santa Cruz Biotechnology, Dallas, TX, USA), and anti-β-actin (1:5000 dilution, Santa Cruz Biotechnology, Dallas, TX, USA) were used as primary antibodies, and the secondary antibodies were conjugated to horseradish peroxidase (HRP).

### 4.7. Serum Stability Testing

BmKDfsin4 was incubated with 10% FBS or ddH_2_O at 37 °C for 4 h, 8 h, 12 h and 24 h. Then, BmKDfsin4 was added to HepG2.2.15 cells at 10 μM and the cells were incubated at 37 °C in 5% CO_2_ for 48 h. The concentrations of HBeAg and HBsAg were detected using ELISA, and the antiviral activity was then evaluated.

### 4.8. Statistical Analysis

Data are shown as mean ± standard deviation from at least three separate experiments. Statistical significance was tested using a two-tailed Student’s *t*-test. IC_50_, CC_50_ and HC_50_ were calculated using the Statistical Product and Service Solution (SPSS) software (Version 21, IBM, Armonk, NY, USA).

## Figures and Tables

**Table 1:** Pharmacological profiles of BmKDfsin4 against HBeAg, HBsAg and HBV DNA on HepG2.2.15 cells.

Peptide	Viral Index	IC_50_ (μM) ^1^	HC_50_ (μM) ^2^	CC_50_ (μM) ^3^	SI ^4^
BmKDfsin4	HBeAg	3.95	66.85	167.82	42.49
	HBsAg	2.28			73.61
	HBV DNA	1.26			133.20

^1^ The IC_50_ (50% inhibitory concentration) value of HBV viral index was determined in HepG2.2.15 cells; ^2^ The HC_50_ (50% hemolysis concentration) value was determined as described above; ^3^ The CC_50_ (50% cytotoxic concentration) value of HepG2.2.15 cells was determined using the MTT assay; ^4^ The SI (select index) was calculated using the ratio CC_50_/IC_50_.

## Supplementary Materials

The following are available online at www.mdpi.com/2072-6651/8/5/124/s1, Table S1: Scorpion derived candidate antimicrobial peptides and their derivative peptides.
