# Supplementary material for: A Scorpion Defensin BmKDfsin4 Inhibits Hepatitis B Virus Replication in Vitro
Source: Toxins (Basel). 2016 Apr 27;8(5):124. doi: 10.3390/toxins8050124 (PMC4885039; doi:10.3390/toxins8050124)
Supplement: Supplementary file 1 [file toxins-08-00124-s001.pdf]

Supplementary Materials: A Scorpion Defensin BmKDfsin4 Inhibits Hepatitis B Virus Replication *in Vitro*

Zhengyang Zeng [1], Qian Zhang [1], Wei Hong [1], Yingqiu Xie [2], Yun Liu [1], Wenxin Li [1], Yingliang Wu [1] and Zhijian Cao [1,*]

Table S1. Scorpion derived candidate antimicrobial peptides and their derivative peptides.

Peptide	Amino Acid Sequence	Length
109261	FIGAIARLLSKIF	13
109262	LFRLIKSLIKRLVSAFK	17
153263	FIKAIARLLRKIF	13
155591	SLIGGLVSAFK	11
183196	LWGEIWNTVKGLI	13
183197	GFLGNLWEGIKTAL	14
183198	IWGALLSGVADLL	13
183199	GILDALTGIL	10
183200	GILDAITGLL	10
183202	FDLGGLIKGVVDLF	14
183211	GIADILKGLL	10
183214	FLWSLIPSAISAVTSLIKK	19
183215	FLVGILPRMRGFITPFLKKVR	21
183218	FLFNVIPHAINATASLIKK	19
BmKn2-T1	FIKAIARLLSKIF	13
BmKn2-T2	FIKAIASLLSKIF	13
BmKn2-T3	FIKAIWSGLSKIF	13
BmKn2-T5	FIKAIWRGLNKIF	13
BmKn2-M1-1	FIRIARLLRKIF	12
BmKn2-M1-2	FIRIRLLRKIF	11
BmKn2-M1-3	FIRIARLLRIF	11
BmKn2-M1-4	FIRIRLLRIF	10
BmKn2-M1-6	FIRIRLRIF	9
BmKDfsin4	GFGCPFNQGQCHKHCQSIRRRGGYCDGFLKTRCVCYR	37
Hp1473-1	IFKAIWSRINRLF	13
